# Recent Advances in Image-Guided Locoregional Therapies for Primary Liver Tumors

**DOI:** 10.3390/biology12070999

**Published:** 2023-07-13

**Authors:** Cody R. Criss, Mina S. Makary

**Affiliations:** 1 OhioHealth Riverside Methodist Hospital, Columbus, OH 43214, USA; cc917811@ohio.edu; 2 Department of Radiology, The Ohio State University Medical Center, Columbus, OH 43210, USA

**Keywords:** primary liver cancer, hepatocellular carcinoma, imaging, locoregional therapy, transarterial chemoembolization, radioembolization

## Abstract

**Simple Summary:**

Primary liver cancer is the third most common cause of cancer-related deaths worldwide. Risk factors for primary liver cancer include chronic viral hepatitis B and C infections, alcohol abuse, non-alcoholic fatty liver disease, and obesity. Surgical resection and/or transplantation is the mainstay treatment for candidates with primary liver tumors. However, minimally invasive, image-guided locoregional therapies have become an integral part of liver cancer treatment and management, depending on staging. In this manuscript, the authors provide a comprehensive overview of the antineoplastic mechanisms underpinning locoregional therapies and the current state of the literature on the efficacy of these therapies for primary liver cancer. We also discuss emerging advances in treatment, such as the adjuvant use of immunotherapies and molecular targeting agents with locoregional therapy. This review highlights the emerging technological advancements and image-guided procedures used to treat primary liver cancer.

**Abstract:**

Primary liver cancer is the leading cause of cancer-related deaths worldwide. with incidences predicted to rise over the next several decades. Locoregional therapies, such as radiofrequency or microwave ablation, are described as image-guided percutaneous procedures, which offer either a curative intent for early-stage hepatocellular carcinoma or bridging/downstaging for surgical resection or transplantation. Catheter-driven locoregional therapies, such as transarterial chemoembolization and radioembolization, induce tumor hypoxia, can be palliative, and improve survival for early-to-intermediate hepatocellular carcinoma and unresectable intrahepatic cholangiocarcinoma. Herein, we provide a comprehensive overview of the antineoplastic mechanisms underpinning locoregional therapies, different treatment approaches, and the current state of the literature for the efficacy of locoregional therapies for primary liver cancer. We also discuss emerging advancements, such as the adjuvant use of immunotherapies and molecular targeting agents with locoregional therapy, for the treatment of primary liver cancer.

## 1. Introduction

Liver cancer constitutes one of the most common causes of malignancy worldwide, and rates for primary liver tumors are steadily rising in the United States [1,2]. The highest reported cases of liver cancer are in Eastern Asia and Middle Africa, and the incidence in men is roughly 2–4 times that of women [1]. Perhaps most alarmingly, liver cancer carries a high risk of mortality, with a 5-year survival rate of 6.5% [1]. Major risk factors have been identified for primary liver cancer, including chronic viral hepatitis B and C infections, alcohol abuse, non-alcoholic fatty liver disease, and obesity. There are two main types of primary liver cancers, including hepatocellular carcinoma and intrahepatic cholangiocarcinoma. In general, surgical resection and/or transplantation is the mainstay treatment for candidates with primary liver tumors. However, locoregional therapies, defined as minimally-invasive, image-guided procedures, have become an integral part of liver cancer treatment and management [3,4,5]. Depending on staging, image-guided locoregional therapies (iLRT), such as ablation (e.g., radiofrequency ablation, microwave ablation, cryoablation), transarterial embolization (TAE) or chemoembolization (TACE), or radioembolization (TARE) can be used as curative, neo-adjunctive, or palliative treatment regimens. The efficacy of these techniques is evaluated by follow-up imaging, via CT or MRI, and the gold standard tool for assessing treatment responses to these techniques is the Response Evaluation Criteria in Solid Tumors (RECIST) [6,7]. It is widely recognized that changes in tumor size, as measured by RECIST, can be used as surrogate endpoints for survival length, meaning that improvements in tumor size often correlate with longer survival times [7]. Within the following manuscript, we provide a brief overview of primary liver cancers and describe the treatment approaches of iLRT, the rationales for treatment, and new emerging evidence for their use.

## 2. Primary Liver Cancers

### 2.1. Hepatocellular Carcinoma

Hepatocellular carcinoma (HCC) is the sixth most common cancer worldwide, accounting for 80–90% of primary liver cancer cases [8,9]. The highest incidence rates are seen in sub-Saharan Africa and Southeast Asia, where viral risk factors, such as hepatitis B virus (HBV), are endemic [10]. In developed countries, HCC incidence is rising due to the increasing prevalence of non-alcoholic fatty liver disease, obesity, and diabetes [11]. In the United States, HCC incidence has tripled since the 1980s, accounting for up to 90% of primary liver cancers [12]. The epidemiology of HCC is complex and multifactorial, with risk factors including viral hepatitis, alcohol consumption, metabolic disorders, and exposure to hepatotoxic chemicals [9,10,13].

HCC is a heterogeneous disease with diverse histological subtypes and molecular characteristics. The most common histological subtype is well-differentiated HCC, which accounts for about 30% of cases. Poorly differentiated HCC, also known as hepatoblastoma-like HCC, is a rare, but aggressive, subtype that is associated with worse outcomes [14]. Other histological subtypes also exist, including fibrolamellar, scirrhous, and macrotrabecular-massive subtypes [14,15]. The tumor microenvironment plays a crucial role in HCC development and progression, with chronic inflammation, immune dysregulation, and fibrosis acting as key drivers [14]. Recent advances in molecular profiling technologies have identified new molecular subtypes of HCC, which may have clinical implications for patient stratification and treatment [16].

**Figure 1:**
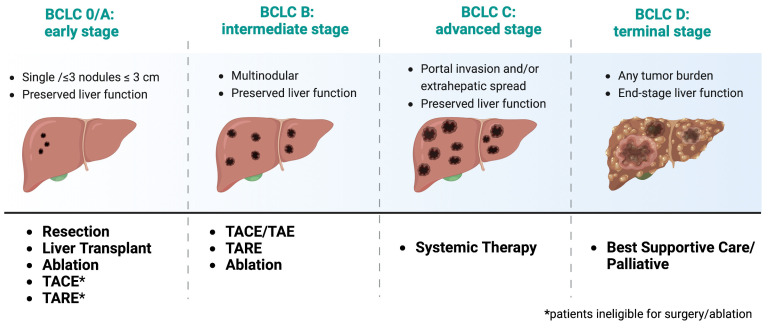
Treatment recommendations based on recent updates from the 2022 Barcelona Clinic Liver Cancer (BCLC) Guidelines [18]. Adapted from “Barcelona Clinic Liver Cancer (BCLC) Staging and Classification”, by BioRender.com (2023). https://app.biorender.com/biorender-templates, accessed on July 2023.

Prognosis of HCC varies depending on several factors, including tumor stage, liver function, and overall health status. HCC is a deadly disease, with a 5-year survival rate of less than 20% [17]. However, early detection and treatment can significantly improve outcomes. The Barcelona Clinic Liver Cancer (BCLC) staging system is widely used to classify HCC patients into different treatment categories (e.g., surgery, iLRT, and systemic treatment) based on tumor burden, liver function, and performance status (Figure 1) [18]. The classification system was recently updated in 2022, and it has been externally validated and endorsed by the Association for the Study of the Liver (EASL) and the American Association for the Study of Liver Diseases (AASLD) [18]. Patients with early-stage HCC (BCLC stage 0 or A) have better outcomes and are eligible for potentially curative treatments, such as surgical resection, liver transplantation, or ablation therapy. However, most patients present with advanced-stage disease (BCLC stage B or C), so they have limited treatment options. For patients with advanced-stage disease, systemic therapy is the standard of care, but the efficacy of these treatments is modest, and there is an urgent need for new therapeutic options. Patients with end-stage HCC (BCLC stage D) are typically managed with supportive care.

### 2.2. Intrahepatic Cholangiocarcinoma

Intrahepatic cholangiocarcinoma (ICCA) is a rare and aggressive cancer arising from the biliary tree within the hepatic parenchymal system [19]. ICCA exhibits traits of cholangiocyte differentiation, and it is likely to originate, mainly, from the epithelial cells that line the bile ducts, known as cholangiocytes [19,20]. Nevertheless, the tumors can also emerge from peribiliary glands and hepatocytes, depending on the location and underlying liver condition. It is the second most common type of primary liver cancer, after HCC carcinoma, accounting for roughly 10–15% of primary liver cancers [21,22]. Reports have also shown progressive increases in the incidence of ICCA worldwide [23,24,25]. However, the epidemiology of ICC remains complex and poorly understood due to its rarity and lack of population-based studies [25].

In the United States, the incidence of cholangiocarcinoma has almost tripled over the past three decades [26]. Similar to HCC, chronic liver disease, including cirrhosis and hepatitis B or C infection, is a significant risk factor for ICCA [19]. Other risk factors include exposure to certain chemicals, such as thorium dioxide and vinyl chloride, as well as inflammatory bowel disease [19,27,28]. There is also a strong association between cholangiocarcinoma and liver fluke parasitic infections within parts of Southeast Asia [19]. Prognosis of cholangiocarcinoma is poor, with a 5 year overall survival rate ranging from 25–31% and a recurrence rate ranging from 40–64% [29,30]. Even worse, ICCA is often beyond the limits of surgical therapy at the time of diagnosis, and the median survival time after treatment with chemoradiotherapy is only 10 months [31]. Surgical resection is the only potentially curative treatment for ICCA, but only a minority of patients are eligible for surgery due to the advanced stage of the disease at the time of diagnosis [32]. Therefore, for unresectable disease, candidates must rely on other non-surgical methods for disease management, such as iLRT or chemotherapy. In the next section, we provide an overview of techniques and current evidence in support of iLRT in the context of primary liver cancer.

## 3. Image-Guided, Tumor-Directed Locoregional Therapies

### 3.1. Rationale for Liver Cancer Treatment

The majority of the liver’s blood supply, about 80%, is received from the portal vein, while only 20% comes from the hepatic artery [33,34]. This division of blood supply has been an important framework for directing the locoregional therapy used to treat HCC. Furthermore, iLRTs have become crucial components to HCC management as curative, adjunctive, and palliative treatment options for individuals who do not qualify for surgery (Table 1). On the other hand, ICCA is less vascular than HCC, suggesting iLRT may not play as significant of a role in treating this type of tumor. Nevertheless, numerous studies have demonstrated interventional iLRTs can provide survival benefits in cases of unresectable ICCA [35]. Although these therapies are generally used for palliative purposes for ICCA, they can also help control the disease (Table 1). However, as mentioned above, studying these methods can be difficult due to the rarity of ICCA combined with the small number of eligible patients for each non-curative treatment method.

### 3.2. Ablation Techniques

**Figure 2:**
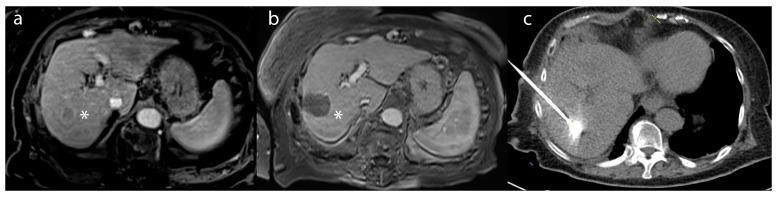
A 55-year-old male patient with (**a**) a lesion in segment 6 biopsy–proven as hepatocellular carcinoma (*)—on post-contrast T1-weighted imaging. (**b**) After microwave ablation, the lesion (*) demonstrated a lack of enhancement compatible with a complete radiographic response on the 1-month follow-up MRI (**c**) intraprocedural treatment CT of microwave ablation.

Ablative therapies include different procedures, such as percutaneous ethanol injection (PEI), radiofrequency ablation (RFA), microwave ablation (MWA), and cryoablation [3,4]. However, the goal of thermal ablation is to use heat extremes to induce tumor death through coagulative necrosis, eliminating undetected cancer microenvironments [37]. The procedure can be performed under moderate sedation or general anesthesia, and it involves the use of a percutaneous probe that navigates to the region of the tumor under CT or MRI guidance. In the context of RFA, the probe delivers frictional high-frequency alternating current to the target tissue, generating heat and, ultimately, the coagulative necrosis of the tumor. Temperatures (50–100 °C) produced by RFA denature proteins, disrupt cellular membranes, and induce thermal coagulation, leading to tumor cell death(Figure 2) [37,38]. After the procedure, patients are usually monitored through multiphasic CT or magnetic resonance imaging (MRI) to evaluate imaging response (Figure 2b). This assessment is typically done 1 month after the procedure. RFA has gained recognition as a well-established therapeutic approach due to its effectiveness, reproducibility, minimal incidence of complications, and widespread accessibility [39]. MVA, which employs electromagnetic energy to induce tumor cell injury, can also be particularly advantageous for liver tumors, due to its enhanced and predictable convection profile, sustained higher intratumoral temperatures, quicker ablation durations, and feasibility of treating multiple lesions, concurrently, using multiple probes [39,40,41].

In general, thermal ablation is used to induce an adequate margin (usually 5–10 mm) around the tumor. If a sufficient margin around the tumor can be achieved, ablation is considered curative [3,5,42]. The efficacy of complete necrosis, after ablation for a single HCC lesion from 2–3 cm, is approximately 90% [38], but its efficacy decreases with larger or later-stage lesions, where undetected microsatellites are often found. For early-stage HCC, meta-analyses of four randomized control trials (RCT) found no differences in all-cause mortality between surgical intervention and radiofrequency ablation [43]. As with HCC, ablation techniques have proven to be a safe and well-tolerated therapeutic approach for ICCA, specifically, in patients harboring small tumors that have not invaded beyond the confines of the bile duct and surrounding tissue and, therefore, may be considered a potentially curative modality [4,35]. Furthermore, due to the highly aggressive and heterogeneous nature of ICCA, many patients with ICCA are not surgical candidates because advanced disease is common at the time of presentation [28]. Patients with unresectable or recurrent ICCA tumors treated with RFA exhibit 1, 3, and 5-year overall survival rates of 82, 47, and 24%, respectively [44]. It has been reported that RFA provides significant improvement in the median overall survival (OS) rates, which range from 20 to 60 months [4]. This stands in stark contrast to the median OS rates of 3–8 months observed in patients with unresectable ICC who did not receive any form of treatment [4]. 

### 3.3. Transarterial Chemoembolization Techniques

**Figure 3:**
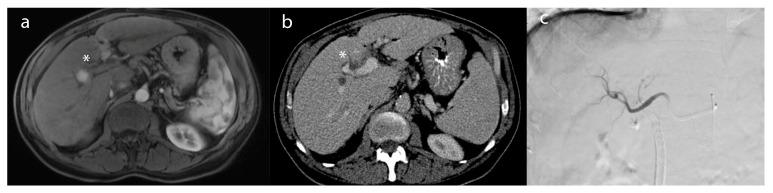
A 62-year-old female patient with (**a**) an arterially-enhancing lesion (*) in segment 8, compatible with hepatocellular carcinoma on post-contrast T1-weighted imaging. (**b**) After TACE, the lesion (*) demonstrated a lack of enhancement, compatible with a complete radiographic response on the 2 month follow-up CT. (**c**) Intraprocedural angiogram of TACE depicting the embolic distribution of the right lobar artery.

The conventional transarterial chemoembolization (cTACE) procedure functions through a distinctive mechanism of action that involves impeding tumor-feeding arteries by injecting chemotherapeutic agents, namely doxorubicin or cisplatin, mixed with the radiopaque contrast agent, lipiodol (Figure 3) [3,42,44,45,46]. The process is intended to limit the supply of nutrients and oxygen to the tumor, thereby causing its necrosis and subsequent shrinkage [46]. This embolic technique works by creating an embolus within the tumor-feeding artery, obstructing the blood flow, and trapping the chemotherapeutic agents within the tumor, leading to a local, sustained release of the chemotherapeutic agents (Figure 3c) [46]. The lipiodol facilitates the visualization of the infused agents under fluoroscopy and CT imaging, thus aiding in the accurate delivery of the embolic agent to the targeted area. The procedure itself takes approximately 1–2 h, and patients are typically monitored overnight before being discharged the following day. Incorporating drug-eluting beads, designated as DEB-TACE, has become increasingly popular in numerous medical centers, as it employs embolic microspheres or beads containing chemotherapy drugs [47]. Among the benefits of DEB-TACE over conventional TACE, it enables a steady and regulated administration of the therapeutic agent, thereby prolonging local exposure to the tumor while minimizing systemic exposure [48,49,50].

TACE is considered first-line therapy for unresectable liver cancer, namely HCC [18,51,52]. The ideal candidates for TACE are patients who have preserved liver function and present with either multinodular or isolated large tumors larger than 3 cm, without any signs of extrahepatic metastasis, vascular invasion, or cancer-related symptoms, and who are not eligible for percutaneous or surgical interventions [18]. TACE has been shown to provide a survival benefit for HCC, as evidenced by a systematic review of 7 randomized control trials yielding an overall improvement in 2-year survival (OR = 0.53 (0.32–0.89); *p* = 0.017) [53]. A large retrospective study found median OS to improve by 6 months with the use of TACE vs. supportive care (8 vs 2 months; *p* ≤ 0.01) [54]. For ICCA, retrospective investigations have shown a statistically significant increase in median survival time for patients receiving TACE, as compared to those who only received supportive treatment (12.2 vs 3.3 months; *p* < 0.001, respectively) [55]. A recent meta-analysis of 11 studies also confirmed the overall survival benefits of TACE for unresectable ICCA compared to supportive treatment [56].

### 3.4. Transarterial Radioembolization Techniques

Transarterial radioembolization (TARE), also known as selective internal radiation therapy (SIRT), was developed under similar technical principles to TACE with the addition of utilizing radioactive beads (e.g., microspheres) that are injected into the hepatic artery under fluoroscopic guidance in order to embolize tumor-supplying vessels [57,58,59,60]. The microspheres are loaded with a beta-emitting isotope, such as yttrium-90 (Y-90), which emits high-energy radiation that causes permanent DNA damage, apoptosis, and destroys cancer cells within the hepatic parenchyma [57]. Unique to TARE, in order to achieve successful radioembolization via adequate cytoreduction and free radical formation, a balance of adequate microsphere coverage and normal oxygen tension to targeted cancer cells is essential. Thus, the process requires appropriately sized particles (20–60 mm) [61,62,63].

Indications for TARE overlap with those of TACE for liver tumor treatment. In a recent update from the BCLC guidelines for image-guided locoregional therapy use, radioembolization has been established as a viable treatment modality for very early-stage (BCLC 0) and early-stage (BCLC A) HCC [18]. These new recommendations are largely based on a 2021 study (Local radioEmbolization using Glass Microspheres for the Assessment of Tumor Control with Y-90 or LEGACY) that investigated the efficacy of radioembolization as a treatment option for early-stage HCC [18,64]. TARE was effective for treating patients with a single HCC tumor measuring less than 8 cm and a preserved performance status. The study reported an objective response rate of 88.3% and median overall survival (OS) of 57.9 months [64]. In fact, a small randomized control trial of patients with HCC (BCLC-A or BCLC-B) showed a longer total time to progression for TARE (*n* = 24; >26 months) compared to cTACE (*n* = 21; 6.8 months) [65]. Data supporting TARE for ICCA are also promising, albeit mostly retrospective, studies with small sample sizes. Outcomes between TACE and TARE are similar (14.2 vs 13.5 months), with no appreciable differences at 2 years [66].

### 3.5. Combining Image-Guided Locoregional Modalities

In recent years, an increasing volume of literature has emerged endorsing the practice of integrating various locoregional therapeutic modalities. This approach is intended to produce a synergistic effect, resulting in enhanced treatment efficacy and improved therapeutic responses. There have been several rationales behind the etiology of why combining thermal ablation may be synergistic. For example, obstructing the hepatic artery and ceasing blood flow in the target zone via embolization can increase the lethal thermal coagulation zone by reducing the tissue cooling due to perfusion, which is coined as “heat sink” [67]. Secondly, a larger volume of sublethal hyperthermia is exposed to high concentrations of the chemotherapeutic agent. This hyperthermic exposure leads to increased cellular membrane permeability, improved intratumoral accumulation of chemotherapy, and increased sensitivity of cytotoxic drugs [67,68,69]. The resulting increase in the volume of coagulative necrosis, including the lethal and sublethal hyperthermic zones, leads to the widening of the ablation margin, which ultimately improves local control by destroying microscopic satellite lesions that are adjacent to the central tumor [67,69,70]. A combination therapy that has garnered significant research attention is the integration of RFA and TACE. Several meta-analyses have demonstrated that this dual approach can enhance overall survival rates beyond those achievable by monotherapy, without incurring any discernible changes in associated complication rates [71,72]. To our knowledge, no studies to date have sought to determine the efficacy of multimodality image-guided locoregional therapy approaches on ICCA outcomes. Nevertheless, based on existing evidence, it does appear that multimodal therapies have a possible advantage, in terms of survival, for primary liver cancer.

## 4. Locoregional and Immunological Therapies

### 4.1. Immunological Basis of Image-Guided Tumor-Directed Therapies

The liver’s diverse cellular composition, including myeloid cells and lymphocytes, makes its immune microenvironment complex. This microenvironment suppresses anti-tumor activity and is a significant obstacle to treating HCC, which is an immunogenic tumor that develops in an immune-suppressed environment. For example, the liver contains macrophages, called Kupffer Cells, as well as T-regulatory and myeloid-derived suppressor cells [73,74]. In the setting of HCC, immune cell activity can be increased, and it can inhibit T-cell cytotoxicity, as well as immune suppression. Immunodysregulation among certain key cells, such as mature dendritic cells and tumor-associated macrophages has also been identified to correlate with poor prognosis [75,76,77].

Several pieces of evidence suggest that iLRT not only directly impact tumor cells but also exerts an immune modulation effect, which may clarify their increased effectiveness when used in combination with immunotherapies [76,78]. Mouse models have shown that animals treated with RFA exhibit increased dendritic cell-related antitumor T-cell immune responses and tumor regression [79]. There has also been evidence that, in addition to inducing thermal coagulative necrosis, RFA can increase heat shock protein expression in the surrounding zone and activate concomitant CD4^+^ and CD8^+^ T-cell effector responses [80,81]. CD4^+^ T-cell and cytokine activations have also been observed after MVA treatment [82]. Apart from activating T-cells, ablation can also regulate anti-tumor immunity by inhibiting myeloid-derived suppressor cells, which correlates with improved recurrence-free survival [76,83,84].

Evidence also supports TACE as a treatment for modulating innate and adaptive immunity. Intra-arterial chemoembolization delivery can lead to the release of cellular debris, pro-inflammatory cytokines, and danger-associated molecular patterns. This triggers a priming effect on adaptive immunity [85]. A prospective investigation of 79 patients with HCC found higher levels of T helper cells 1 month after TACE (*p* = 0.036) [86]. An investigation analyzing the peripheral blood of 114 patients with HCC showed a marked increase in programmed cell death protein 1, which was also associated with improved prognosis [87]. A handful of investigations have also observed changes in immune responses to Y90 radioembolization for HCC and ICCA [88,89,90].

### 4.2. Combining Image-Guided Therapies with Immunotherapy

The observed immunological changes following iLRT have sparked a burgeoning interest in augmenting the efficacy of locoregional therapy through the implementation of combination regimens involving immunotherapy agents [78]. A majority of these investigations have been done in the context of HCC. Sorefanib, a tyrosine kinase inhibitor, has long been considered a salvage systemic therapy for advanced HCC [18]. Prospective, multi-center investigations have highlighted statistically improved progression-free survival for combined TACE + sorafenib (25.2 months) vs. monotherapy (13.5 months) [91]. The meta-analysis has also supported increased time to progression for combination therapy, but it did not identify differences in overall survival [92]. Several studies have also explored other kinase inhibitors (i.e., brivanib and orantinib) with TACE, but they have failed to meet primary overall survival endpoints [78]. Other immunotherapies include programmed death protein-1 (PD-1) and cytotoxic T-lymphocyte-associated protein 4 (CTLA-4) inhibitors. Randomized control trials, exploring the role of (neo)adjuvant immunotherapies in concert with RFA, are currently underway [78]. Observational investigations, combining CTLA-4 inhibitors (i.e., Tremelimumab) and TACE for patients with advanced HCC and hepatitis C, have exhibited a resultant reduction in viral load and an increase in intratumoral CD8+ cells from tumor biopsies [93]. A phase 1 clinical trial is underway using Tremelinumab in combination with radiofrequency ablation or TACE [94]. PD-1 inhibitors, such as Lenvatinib, are also being explored for unresectable HCC. A prospective investigation showed combination therapy with TACE at a higher objective response rate (67.9% vs. 29.6%, *p* < 0.001) and overall survival period (23.9 vs. 15.3 months, *p* < 0.001) [95]. Although still in its infancy, these efforts to enhance the efficacy of iLRT and enhance anti-tumor immune response display promising results.

## 5. Discussion and Future Directions

Primary liver cancers are highly aggressive and, often, fatal diseases that affect millions of people around the world. Considering the heterogeneity of liver cancers and the various prognostic factors that must be considered when determining treatment eligibility, image-guided therapies represent distinctive and pioneering modalities for managing HCC and ICCA. Thermal ablation, TACE, and TARE are all effective locoregional therapies for the treatment of HCC and ICCA. Ablation presents a potentially curative therapeutic option for individuals with early-stage HCC who are not eligible for surgical intervention. Ablation (e.g., RFA or MWA) also improves outcomes for patients with unresectable or recurrent ICCA. Other image-guided therapies, such as chemoembolization, offer improved survival benefits for ICCA and early-to-intermediate-stage HCC. TARE is also a viable treatment modality for early-stage HCC and ICCA, as established by recent guidelines. Although further research is required to investigate and refine the utilization of these tools, they offer a promising, minimally-invasive approach for managing and enhancing outcomes in patients with complex or arduous liver diseases. The combination of different locoregional therapies may produce a synergistic effect, resulting in enhanced treatment efficacy and improved therapeutic responses. These therapies also exert an immune modulation effect, making them candidates for combination with immunotherapies.

The identification of specific immune and molecular changes also offers potential for future developments in disease monitoring. For example, the alpha-fetal protein (AFP) has been long considered a prognostic and treatment response serum biomarker, for surgery and locoregional therapy, in patients with HCC [96,97,98]. Serum AFP response to iLRT has been shown to stratify the risk of HCC recurrence following a liver transplant [99]. Currently, the most commonly utilized biomarkers for the detection and monitoring of ICCA include a carbohydrate antigen (CA19-9) and a carcinoembryonic antigen (CEA) [100]. Given that iLRT can induce immune and molecular modulating effects, such as with CD4^+^, CD8^+^ T cells, and T regulatory cells, it may offer an additional circulating biomarker to monitor treatment response [101]. For example, increased levels of T helper cells post-TACE are associated with greater OS (*p* = 0.007) [86]. As such, in combination with imaging, such as dynamic CT and MRI [102], immunological and molecular biomarkers offer new monitoring methods for treatment response [76,101]

## 6. Conclusions

Primary liver cancers are highly aggressive and often fatal diseases that affect millions of people around the world. Considering the heterogeneity of liver cancers and the various prognostic factors that must be considered when determining treatment eligibility, image-guided therapies represent distinctive and pioneering modalities for managing HCC and ICCA. Ablation presents a potentially curative therapeutic option for individuals with early-stage HCC who are not eligible for surgical intervention. Ablation (e.g., RFA or MWA) also improves outcomes for patients with unresectable or recurrent ICCA. Other image-guided therapies, such as chemoembolization and radioembolization, offer improved survival benefits for ICCA and early-to-intermediate-stage HCC. Although further research is required to investigate and refine the utilization of these tools, they offer a promising, minimally invasive approach for managing and enhancing outcomes in patients with complex or arduous liver diseases.

## Figures and Tables

**Table 1:** Image-guided locoregional therapies and their clinical utility.

iLRT Modality	Procedure Technique	Image Modality Utilized	HCC Clinical Indications	ICCA Clinical Indications
Ablation	Percutaneous probe delivering high-frequency (RFA), microwave (MVA), cooling (Cryoablation), or chemical injection (PEI) directly to tumor cells.	Ultrasound or CT	Curative for non-surgical candidates with very early (BCLC-0) and early (BCLC-A) stage [18]	Bridging/downgrading and palliative for unresectable ICCA (stage III-IV) [35,36]
TACE	Catheter-driven chemotherapy (e.g., doxorubicin), embolic agent and, contrast directly to tumor-feeding vessels.	Angiography	Bridging/downgrading and disease control for early (BCLC-A) and Intermediate (BCLC-B) stage [18]	Bridging/downgrading and palliative for unresectable ICCA (stage III-IV) [35,36]
TARE	Catheter-driven microspheres loaded with Yttrium-90 labeled isotopes that emit β-radiation to tumor-feeding vessels	Angiography	Early (BCLC-A) and Intermediate (BCLC-B) stage [18]	Disease control, Bridging/downgrading and palliative for unresectable ICCA (stage III-IV) [35,36]

RFA, radiofrequency; MVA, microwave ablation; PEI, percutaneous injection; CT, computer tomography; BCLC, Barcelona Clinic Liver Cancer.

## Data Availability

No new data were created or analyzed in this study. Data sharing is not applicable to this article.
